# Histologic and spectroscopic study of pluripotent stem cells after implant in ocular traumatic injuries in a murine model

**DOI:** 10.1186/scrt509

**Published:** 2014-10-20

**Authors:** Gustavo Jesús Vázquez-Zapién, Marlon Rojas-López, Raúl Jacobo Delgado-Macuil, Luis Rubén Martínez-Nava, David Guillermo Pérez-Ishiwara, Mónica Maribel Mata-Miranda

**Affiliations:** Embryology Laboratory, Escuela Médico Militar, Universidad del Ejército y Fuerza Aérea, Cerrada de Palomas S/N, Lomas de Sotelo, Delegación Miguel Hidalgo, México, DF 11200 Mexico; CIBA-Tlaxcala, Instituto Politécnico Nacional, Tepetitla, Ex-Hacienda San Juan Molino Carretera Estatal Tecuexcomac, Tepetitla Km 1.5, Tlaxcala, 90700 Mexico; Research Multidisciplinary Laboratory, Escuela Militar de Graduados de Sanidad, Universidad del Ejército y Fuerza Aérea, Cerrada de Palomas S/N, Lomas de Sotelo, Delegación Miguel Hidalgo, México, DF 11200 Mexico; Programa Institucional de Biomedicina Molecular, ENMyH-IPN, Guillermo Massieu Helguera, No. 239, Fracc. La Escalera, Ticomán, México, DF 07320 Mexico; Cell Biology Laboratory, Escuela Médico Militar, Universidad del Ejército y Fuerza Aérea, Lomas de Sotelo, Delegación Miguel Hidalgo, Cerrada de Palomas S/N, México, DF 11200 Mexico

## Abstract

**Introduction:**

Ocular trauma is defined as a trauma caused by blunt or penetrating mechanisms on the eyeball and its peripheral structures, causing damage with different degrees of affection with temporary or permanent visual function compromise. Ocular trauma is a major cause of preventable blindness worldwide; it constitutes 7% of all corporal injury and 10% to 15% of all eye diseases. Regenerative medicine research has opened up the possibility to use stem cells as a source of cell replacement, so that experimental studies on embryonic stem cells and bone marrow stem cells have been carried out. In this study, we analyzed the histopathological and spectroscopic changes in ocular tissue with trauma, treated with mouse pluripotent stem cells.

**Methods:**

Firstly, mouse embryonic stem cells were seeded. Subsequently, the obtained cells were implanted in a murine model of scleral and retinal damage at the first, second, and fourth weeks post-trauma. At week 12 post-trauma, the eyes were enucleated for histopathologic study (inflammatory response and histological integrity) and spectroscopic analysis by Fourier transform infrared spectroscopy in the attenuated total reflection configuration. Data were analyzed by one-way analysis of variance.

**Results:**

Histopathological results showed that the experimental groups treated with stem cells presented a decrease in the inflammatory response, and the histological integrity was restored, which contrasted with the experimental group treated with saline solution. Moreover, in the spectroscopic analysis, characteristic bands of biological samples were observed in all tissues, highlighting in healthy tissues the presence of C = O bond at 1,745 cm^-1^, which was not observed in the injured and treated tissues. Also, the absorption spectrum of the tissues treated with embryonic stem cells showed bands whose intensity was high at around 1,080 to 1,070 cm^-1^. It has been reported that these bands are characteristic of pluripotent stem cells.

**Conclusions:**

The implant of embryonic stem cells could be a useful therapeutic treatment after traumatic eye injuries or many other eye diseases to reduce the inflammatory response and restore histological integrity. Furthermore, the spectroscopic technique could be used as a complementary technique for detecting stem cell incorporation into various tissues.

## Introduction

“Ocular trauma” (OT) is defined as trauma caused by blunt or penetrating mechanisms on the eyeball and its peripheral structures, causing tissue damage with different degrees of affection with temporary or permanent visual function compromise [1]. This is a worldwide cause of visual morbidity and is a leading cause of non-congenital monocular blindness in children [2].

OT is a major cause of preventable blindness worldwide; it constitutes 7% of all corporal injury and 10% to 15% of all eye diseases. It has become the most frequent cause of hospitalization of ophthalmological patients [3]; in the US, the incidence is almost 2.5 million per year [4].

World Health Organization estimates, in its prevention of accidents program, that there are 55 million eye injuries annually, of which 200,000 are open globe injuries [1]. It is reported that worldwide 1.6 million people are blind as a result of ocular injuries, 2.3 million with low visual acuity bilaterally, and 19 million with low vision or monocular blindness [5].

An open OT should be urgently operated [1]. Closure of OT wounds by penetration must restore the anatomy and functional architecture [6].

Owing to surgical complications, different ways to restore retinal degeneration through some type of transplant have been thought, but, in contrast to solid organ transplants, which only require re-anastomosis of large vessels and ducts, the transplantation of a whole eye would require the restoration of more than a million axonal connections between the inner retina and the lateral geniculate nucleus of thalamus, located several centimeters away [7].

Current medical research has focused mainly on developing therapeutic strategies for neuroprotection and cell replacement. Cell replacement is a novel therapeutic approach to restore visual capabilities on degenerated retinal illness and represents an emerging field of regenerative neurotherapy. Since the discovery of stem cells (SCs), these have been used as a source of cell replacement, so that experimental studies on neural SCs, embryonic SCs (ESCs), and bone marrow SCs are carried out. These studies try to confirm the potential of SC transplantation and the integration in the retina after the transplantation, leading to appropriate visual processing [8, 9].

An SC is defined as a cell capable of dividing indefinitely and differentiating into several specialized cell types, not only morphologically but also functionally. According to their origin and developmental potential, SCs are classified as totipotent, pluripotent, multipotent, and unipotent. With this, we can mention two important SC applications: first, their differentiation potential would allow us to use them to regenerate damaged or destroyed tissue; second, the SCs may be used as a gene therapy vehicle in the case of monogenic diseases such as hemophilia or even as an antitumor vehicle or antiangiogenic therapies [7]. For SC therapies, the retina has the optimal combination of ease of surgical access and an ability to observe transplanted cells directly through the clear ocular media [10].

ESCs have been used in retinal vascular disease, Stargardt disease, retinitis pigmentosa, macular degeneration, and photoreceptor dystrophy with different methods [10–12]. Furthermore, two clinical trials for SC-based therapies in retinal diseases have been approved by the US Food and Drug Administration and initiated by Advanced Cell Technology (Santa Monica, CA, USA), which plans to include patients with Stargardt disease and geographic atrophy secondary to age-related macular degeneration (AMD) [12].

Some studies show the absence of side effects from SC therapy in ocular lesions, such as hyperproliferation, tumorigenicity, ectopic tissue, or rejection, after 4 months of follow-up [13].

In addition to the OT histopathological studies, the potential of infrared vibrational spectroscopy in monitoring biochemical changes associated with retinal response to light incident has been shown [14]. Moreover, the recent adoption of vibrational spectroscopic approaches to study SC differentiation has emerged as a modern technique [15]. One of these modalities, Fourier transform infrared (FTIR) spectroscopy, has been used for the recent characterization of pluripotent and multipotent SCs [16].

The aim of this study is to determine the ability of ESCs to restore eye injuries; histopatological and spectroscopic studies were performed by FTIR, which allowed us to determine the capacity to restore eye tissue after OT.

## Materials and methods

### Embryonic stem cell culture

The mouse embryonic SCs (mESCs) (ATCC, Manassas, VA, USA; SCRC-1011) were seeded at a density of 50,000 cells per cm^2^ on a monolayer of mouse embryonic fibroblasts which were previously mitotically inactive for mESC growing. We used mESC basal medium (ATCC, catalog number SCRR-2010) supplemented with 15% fetal bovine serum, 0.1 mM 2-mercaptoethanol (Invitrogen, Carlsbad, CA, USA; catalog number 21985023), and 1,000 U/mL mouse leukemia inhibitory factor (Chemicon, now part of EMD Millipore, Billerica, MA, USA; catalog number ESG1107). The culture dishes were incubated at 37°C in a humidified 5% CO_2_ and 95% air incubator [17]. When the cultures reached 70% confluency, doses of 20,000 and 50,000 mESCs were obtained and resuspended in 0.025 mL of 0.09% balanced salt solution (BSS).

### A murine model of ocular trauma

This experimental work followed the guidelines of the Norma Oficial Mexicana Guide for the use and care of laboratory animals (NOM-062-ZOO-1999) and for the disposal of biological residues (NOM-087-ECOL-1995). The animals were males of the *Rattus norvegicus* strain and were 12 weeks of age. They were kept in metabolic cages in a temperature-controlled environment with a 12-hour light/dark cycle and were allowed free access to food and water at all times.

Since there are no reports of mouse models with OT, this model was standardized by injuring the sclera and the entire retina. By intraperitoneal injection, anesthesia with ketamine (50 mg/kg) and xylazine (8 mg/kg) was administered by using asepsis and antisepsis protocols with 10% povidone-iodine solution. OT was performed by scleral puncture by the pars planar with a 27G trocar, reaching the retina and causing a lesion of 1 mm in length in the temporary area outside the vascular arcades in order to avoid vascular damage and to obtain a suitable visual field for the realization of the lesion. The total thickness of the retinotomy spanned the internal limiting membrane and the photoreceptors layer. These procedures were performed by an ophthalmologist using an indirect ophthalmoscope and a 22-diopter lens. All procedures involving animals were approved by the Ethics Committee for Animal Research of Escuela Militar de Graduados de Sanidad.

### Implantation of mouse embryonic stem cells (pluripotent)

**Figure 1:**
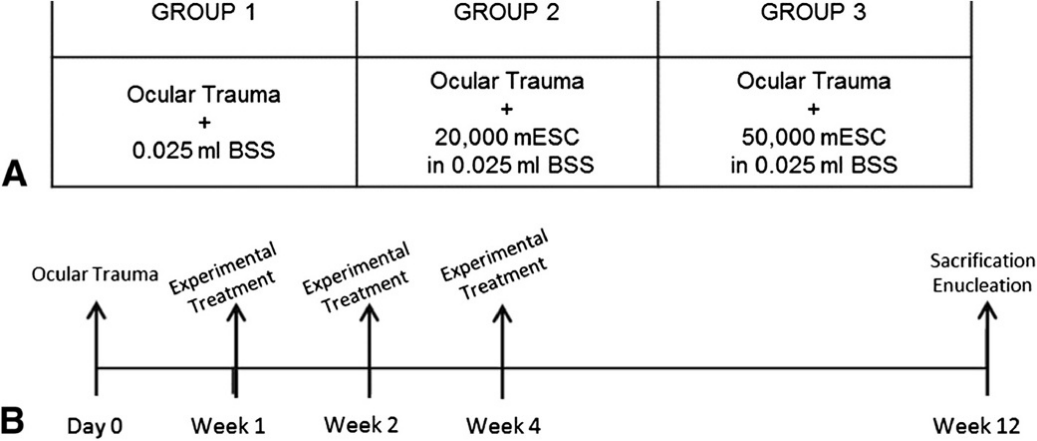
**Experimental methodology. (A)** Experimental groups. To all groups, an ocular trauma (OT) was induced; group 1 received balanced salt solution (BSS), group 2 had an intravitreal implant of 20,000 mouse embryonic stem cells (mESCs) resuspended in 0.025 mL of BSS, and group 3 was treated with an intravitreal implant of 50,000 mESCs resuspended in 0.025 mL of BSS. **(B)** Experimental methodology. After OT (day 0) and at the first, second, and fourth weeks post-trauma, the groups received their experimental treatment according to the group that they belong to; at 12 weeks post-trauma, rats were sacrificed and the eyes were enucleated for their histopatological and spectroscopy analysis.

In this study, 30 male *Rattus norvegicus* rats were randomly divided into three groups (n =10 right eyes of rat per group). All members of the groups were subjected to the OT described above and subsequently received the intravitreal injections with different treatments according to the group that they belonged to at the first, second, and fourth week post-trauma. Group 1 (control group) had intravitreal injections of 0.025 mL of BSS, group 2 received an intravitreal implant of 20,000 mESCs resuspended in 0.025 mL of BSS, and group 3 had an intravitreal implant of 50,000 mESCs resuspended in 0.025 mL of BSS (Figure 1).

### Histopathological and ATR-FTIR spectroscopy analysis

After 12 weeks of OT and treatment, the animals were sacrificed and their eyes stored at -40°C; thereafter, histological sections of 4-μm thickness were obtained by using a cryostat (Jaelsa, Madrid, Spain). For the histopathological study, the histological sections were immediately fixed in 4% paraformaldehyde and stained with hematoxylin and eosin in order to be analyzed by a pathologist using brightfield microscopy (Carl Zeiss, Jena, Germany; Axioskop 2 plus). An FTIR spectrometer (model Vertex 70; Bruker, Billerica, MA, USA) with attenuated total reflectance (ATR) was used for vibrational analysis, and each tissue section was placed directly onto the surface of the ATR crystal spectrometer.

## Results

**Figure 2:**
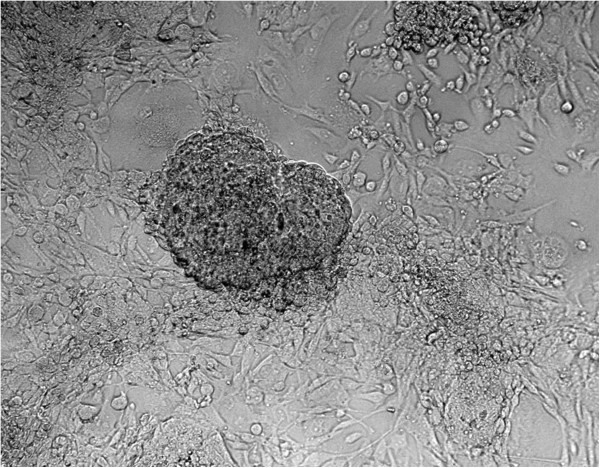
**Stem cell culture.** Mouse embryonic stem cells (400×) integrating a colony that is under a mouse embryonic fibroblast monolayer.

The mESCs are adherent cells characterized mainly by their growth in colonies (Figure 2). A typical colony formation was shown 24 hours after seeding, and the culture reached confluence after 72 hours of culture.

### Histology of the ocular trauma in a murine model

**Figure 3:**
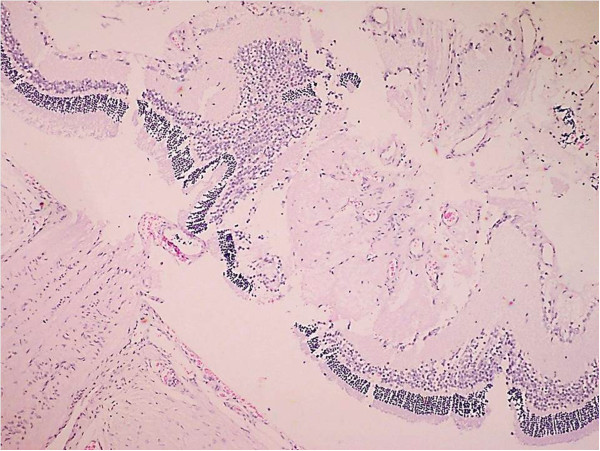
**Histology of the ocular trauma in a murine model.** Alteration of the normal histological conformation of the retina characterized by dissemination of the retinal neuron nucleuses 48 hours after ocular trauma (400×).

Forty-eight hours after OT in the mouse model, we noted destruction of most of the retinal layers, altering the normal histological conformation characterized by dissemination of the retinal neuron nucleuses (Figure 3).

### Inflammatory response and histological integrity

Intravitreal applications were carried out at a set time and were uneventful; rejection, endophthalmitis, or any other secondary complications such as macroscopic damage were not observed. The inflammatory response was graded by using as a criterion the number of lymphocytes as follows: nil, mild (<5 cells per field of view), moderate (≥5 cells per field of view), and severe (≥10 cells per field of view). Furthermore, grades of histological integrity were established as follows: restored, mild alteration, moderate alteration, and severe alteration, according to the presence of fibroblastic reaction, loss of the longitudinal axis of the fibroblasts or fibrocytes, and recapilarization. In addition, specifically in scleral tissue, the arrangement of the collagen fibers and the displacement of normal tissue structures were analyzed, whereas in the retina, the architecture and cellularity of retinal neurons were examined. However, it is important to mention that none of the experimental groups showed any signs of severe inflammatory response or severe alterations of the histological integrity.

**Figure 4:**
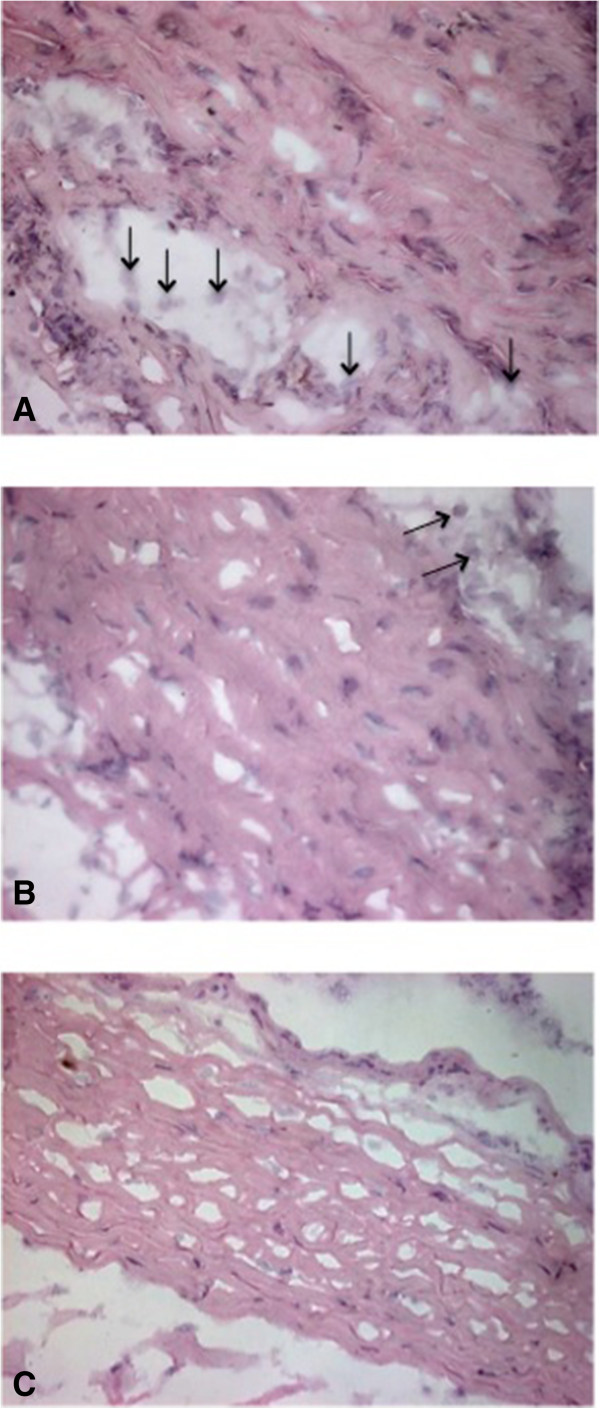
**Scleral inflammatory response and histological integrity.** Scleral tissues (400×). **(A)** In group 1, moderate inflammatory response characterized by the presence of lymphocytes (↑) and loss of the arrangement of the collagen fibers are shown. **(B)** In group 2, few lymphocytes (↑) and a slight alteration in the histological integrity were observed. **(C)** In group 3, cells involved in the inflammatory response are not shown, and the arrangement of the collagen fibers was restored, which means that the scleral histology was recovered.

**Figure 5:**
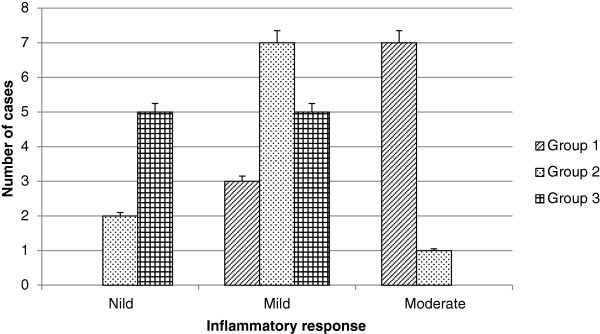
**Inflammatory response.** The inflammatory response was graded as nil, mild, moderate, and severe according to chronic inflammatory cell infiltrate. None of the experimental groups showed any signs of severe inflammatory response. Most members of group 1 (control group) had a moderate alteration, whereas most members of groups 2 and 3 (groups that received mouse embryonic stem cells) showed a nil and mild inflammatory response (*P* <0.0001).

**Figure 6:**
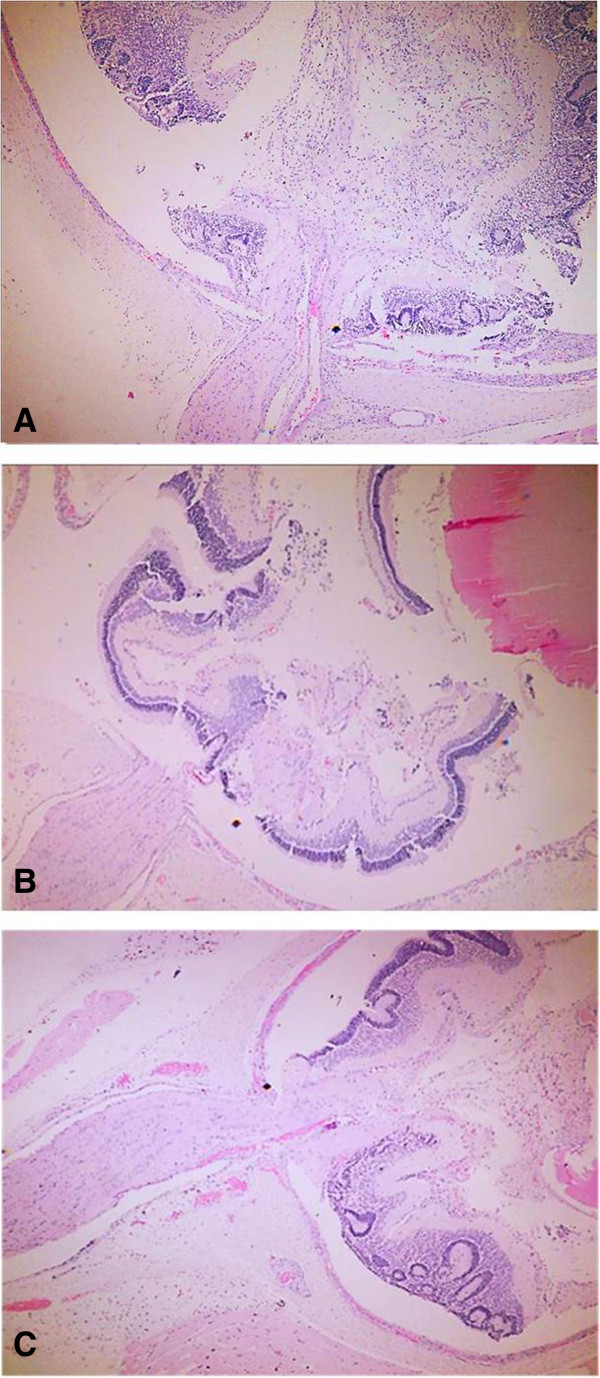
**Retinal histological integrity.** Retinal tissues (100×). **(A)** In group 1, histological integrity, characterized by dissemination of neuronal cell nuclei, was not retained. **(B)** In group 2, restored architecture is observed with homogenization of the retinal layers. **(C)** In group 3, restored architecture with normal neuronal cellularity is shown.

**Figure 7:**
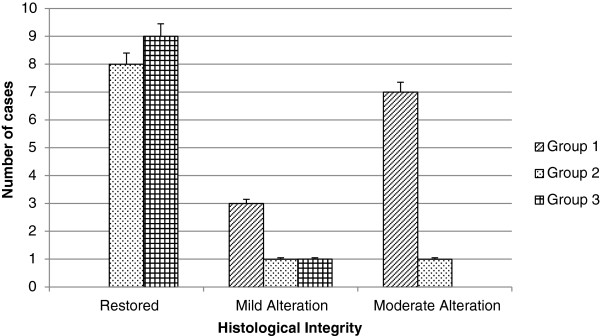
**Histological integrity.** The histological integrity was graded as restored, mild alteration, moderate alteration, and severe alteration, according to the presence of fibroblastic reaction, loss of the longitudinal axis of the fibroblasts or fibrocytes, arrangement of the collagen fibers, displacement of normal tissue structures, and recapilarization. None of the experimental groups showed any signs of severe alterations of histological integrity. Most members of group 1 (control group) had a moderate alteration, whereas groups 2 and 3 (groups that received mouse embryonic stem cells) showed a restored histological integrity (*P* <0.0001).

According to the inflammatory response, it is worth to mention that the density and arrangement of the scleral collagen fibers avoid the diapedesis of a large number of typical inflammatory cells. Consequently, the histological architecture hinders the migration and localization of inflammatory cells in this region.Group 1 showed moderate inflammatory response in the 70% of cases, characterized by the presence of lymphocytes that are localized between the scleral collagen fibers that were displaced after injury (Figure 4A). Seventy percent of group 2 exhibited a mild inflammatory response, and we observed a few lymphocytes in scleral tissue (Figure 4B). In 50% of the specimens in group 3, signs of inflammatory response were not observed (Figure 4C).In the results obtained from inflammatory response, a big difference between group 1 and the groups that received mESC intravitreal implant (groups 2 and 3) was demonstrated. Groups 2 and 3 showed a marked decrease in inflammatory response. Furthermore, the inflammatory response observed in group 3 was even lower compared with group 2 (Figure 5).In regard to histological integrity, 70% of cases in group 1 showed a moderate alteration characterized by an irregular pattern of the collagen fibers in the scleral tissue, which exhibited distortion and rupture. Also reactive fibroblasts and fibrocytes were observed, as well as the decrement of the histological architecture. These histopathological changes correspond to damage by mechanical action (Figure 4A). In the same way, in the retinal region, we observed a modified architecture in a topographic context, characterized by the dissemination of the nucleuses of the retinal neurons (Figure 6A).Furthermore, in group 2, histological integrity was restored in 80% of samples; in the scleral tissue, collagen fibers with a regular pattern are noted and the longitudinal axis is mildly restored (Figure 4B). However, in the retinal area, a restored architecture is observed in its topography, and homogenization of the retinal layers is noted since neuronal nucleuses are clustered in a defined area (Figure 6B).In regard to group 3, 90% of cases restored their histologic integrity. In the scleral tissue, retrieval on the arrangement of collagen fibers is noted in a homogeneous way with a regular geometrizacion pattern in space. Also, a major recapilarization was observed, and fibrocytes recovered their normal longitudinal axis with a very well-defined polarity (Figure 4C). However, in the retinal zone, the architecture was evidently restored up to 90% since neuronal cellularity shows a normal appearance (Figure 6C). In joint analysis, it is evident that groups 2 and 3 had a better histological integrity relative to group 1. As seen in the inflammatory response, histological integrity was restored in a higher proportion in group 3 (Figure 7).

### Infrared absorption in histological sections

**Figure 8:**
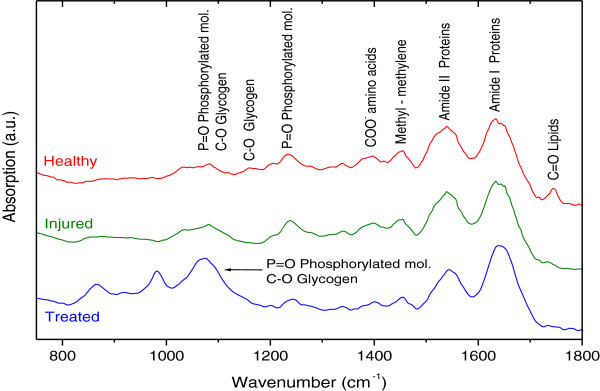
**Infrared absorption spectrum.** Fourier transform infrared spectroscopy in the attenuated total reflection configuration (ATR-FTIR) of representative histological sections in three different conditions: healthy tissue, damaged tissue, and damaged tissue treated with 55,000 mouse embryonic stem cells. a.u., arbitrary units.

Infrared absorption measurements of each representative histological section from each condition (healthy tissue, injury tissue, and injury tissue treated with 50,000 mESCs) were carried out (Figure 8). The presence of many functional groups, characteristic of biological sample, could be observed. First, in healthy tissue sample, C = O bonds were observed at 1,745 cm^-1^, corresponding to ester lipids [14].

The following bands corresponded to amide I (1,635 cm^-1^) and amide II (1,538 cm^-1^) bands of proteins [15]. Likewise, in the three histological sections in different conditions, the methyl and methylene groups from lipids and proteins at 1,454 cm^-1^ were observed [16]. A weak COO-1 absorption band at 1,396 cm^-1^ originating from the amino acid side chains could be shown. The P = O bonds at 1,238 and 1,080 cm^-1^ in healthy and injured tissues are attributed to the phosphodiester groups from phosphorylated molecules and glycogen. Also, the absorption band appearing at 1,158 cm^-1^ was associated with a glycogen CO bond, whereas the bands between 1,080 and 1,070 cm^-1^ were due to the P = O bond of phosphorylated molecules and to the C-O bond of the glycogen [14].

### Statistical analysis

The treatments were randomly assigned. For the two histopathological variables studied (inflammatory response and histological integrity), an ordinal measurement scale was used. Data were analyzed by one-way analysis of variance by using Statistic 8.0. For the two studied variables, the difference between groups 2 and 3 did not reach statistical significance. However, between group 1 and groups 2 and 3, a statistically significant (*P* <0.0001) was shown.

## Discussion

The use of SCs in ophthalmic diseases currently is a promising treatment that is in active research, and SCs have increasingly been employed for experimental studies. However, clinical trials are scarce. In addition, the few existing studies are more focused on retinal vascular diseases such as diabetic retinopathy, degenerative retinopathy, and retinal dystrophies (AMD, retinitis pigmentosa, and Stargardt disease) [18]. Early SC therapy approaches are based on regeneration of damaged tissues such as the retinal pigment epithelium and photoreceptors, and the entire retina replacement with differentiated SCs is a more ambitious goal.

The aim to use SCs in ophthalmic diseases is to replace damaged cells and preserve cell function. Therefore, this study tried to demonstrate the positive effects on histological restoration after the treatment with ESCs in an OT. This kind of trauma affects the scleral and retinal histological integrity, causing decreased vision and sometimes blindness, which severely affects the patient’s quality of life, besides being associating with a profound impact on the economy.

In accordance with our results, we can say that treatment with ESCs may be useful to preserve the scleral and retinal histology and probably the visual function after OT.

In our study, there was no histological evidence of neoplasia (teratomas) at 11 weeks post mESC implantation. In contrast, Arnhold *et al*. [19] and Chaudhry *et al*. [20] reported the formation of teratomas at 6 and 8 weeks after differentiated ESC implantation, respectively. This fact suggests that the implantation of ESCs in a undifferentiated state could have a greater utility. Likewise, we did not identify signs of abnormal growth, hyperproliferation, or immune-mediated organ rejection over 3 months’ follow-up; these results are similar to those reported by Steven *et al.* [13].

Currently, few clinical trials in OT handle pluripotent cells. However, some researchers are performing clinical trials in retinal dystrophies. For example, Bainbridge *et al*. are implanting human ESCs in doses from 50,000 to 200,000 cells in patients over 18 years old with a retinal dystrophy [21].

In this study, we implanted 20,000 and 50,000 cells per dose in different experimental groups without observing a statistically significant difference between the two groups. However, in the group that received doses of 50,000 mESCs, there was not reported a single case with inflammatory response, and histological integrity was recovered in 90% of cases, which would be very clinically useful. Therefore, this study opens the door to future research to assess effectiveness versus cost-effectiveness by using different doses of SCs.

In regard to the results of infrared ATR-FTIR absorption, the presence of bands characteristic of biological samples was observed, encompassing bindings associated with lipids, proteins, phosphorylated amino acid compounds, and mainly carbohydrates.

It is worth noting that the tissue section of healthy sample presented a C = O bond at 1,745 cm^-1^ corresponding to esters lipids. These lipids were not observed in samples of injured and treated tissue, and this was probably due to cell membrane destruction. In addition, in the spectrum of the samples treated with mESCs, we detected intense absorption bands between 1,080 and 1,070 cm^-1^ related to carbohydrates as glycogen, but these were mainly phosphorylated compounds.

Cao *et al*. have reported that one of the vibrational characteristics that distinguish pluripotent SCs with respect to specialized cells is increased intensity of FTIR related with the presence of phosphorylated compound (1,080 to 1,070 cm^-1^) [16]. In this way, the use of this spectroscopic technique is proposed as an additional tool for SC detection and its incorporation to injured ocular tissue.

More randomized controlled clinical trials using SCs in eye diseases are needed, not only for degenerative and vascular diseases or dystrophies but also for any eye pathology in which the anatomy and function are affected, such as the OT, in order to establish a safe dose with greater benefits and to determine whether the use of ESCs is effective in treating a particular condition.

## Conclusions

We have shown in a preliminary way that the mESC implant could be very useful as a therapeutic tool after an OT or many other ophthalmological diseases since this therapy reduces the inflammatory response and restores the histological integrity. However, further studies are needed to determine other parameters such as preservation of functionality, localization and biodistribution of mESCs, therapeutic doses, and long-term follow-up. In the same way, the capacity of FTIR spectroscopy as a complementary technique was demonstrated in order to detect the SC implantation and its incorporation in different tissues.

## Abbreviations

AMD: atrophy secondary to age-related macular degeneration
ATR: attenuated total reflectance
BSS: balanced salt solution
ESC: embryonic stem cell
FTIR: Fourier transform infrared
mESC: mouse embryonic stem cell
OT: ocular trauma
SC: stem cell.
